# Exploring the Potential of Pyroptosis-Related Genes in Predicting Prognosis and Immunological Characteristics of Pancreatic Cancer From the Perspective of Genome and Transcriptome

**DOI:** 10.3389/fonc.2022.932786

**Published:** 2022-06-16

**Authors:** Jing Zhang, Xiaomin You, Dong Kang, Guoxiong Zhou

**Affiliations:** ^1^ Department of Gastroenterology, Affiliated Hospital of Nantong University, Nantong University, Nantong, China; ^2^ Department of General Surgery, Rugao Hospital of Traditional Chinese Medicine, Rugao, China

**Keywords:** pyroptosis, pancreatic cancer, prognostic prediction, risk stratification, immune microenvironment

## Abstract

**Objective:**

To probe into the role of pyroptosis-related genes in pancreatic carcinoma.

**Methods:**

Herein, we conducted a comprehensive bioinformatics analysis to evaluate tumor-immune infiltration and tumor mutation burden, the correlations between PRGs, and microsatellite instability and found that 33 PRGS were up- or down-regulated in PC. Then we built the PPI network, which was downloaded from the STRING database. Using TCGA cohort median risk score, PC subjects from the Gene Expression Composite cohort (GEO) data resource were stratified into two risk categories, with the low-PC risk group harboring a higher overall survival (OS) (P = 0.011). We employed the ssGSEA approach to quantify immune cell abundance in separate risk groups separated by risk signature while assessing variations in immune cell invasion. Chemotherapeutic drugs were retrieved from the Genomics of Drug Sensitivity in Cancer (GDSC) data resource.

**Results:**

Eight prognostic PRG models (CASP4, GSDMC, IL-18, NLRP1, NLRP2, PLCG1, TIRAP, and TNF) were established *via* LASSO Cox regression to estimate the OS of PC subjects with medium-to-high accuracy.

**Conclusion:**

Our study is the first to identify a pyroptotic-related prognostic gene feature for PC, providing more options for the prognostic prediction of PC.

## Introduction

Pancreatic cancer (PC) is a highly malignant tumor of the digestive tract with increased incidence in the recent years (1) along with a poor overall prognosis (2). PC is known as the “king of cancer” not only for its aggressiveness and rapid progression but also for its frequent discovery at an advanced stage of disease for the first time. At this time, radical surgery is uncommon and is not responsive to radiotherapy or chemotherapy. The overall 5-yearsurvival rate was only 8% (3, 4). However, when many patients are first diagnosed with pancreatic cancer, it has already advanced, the untreated median survival time is about 6 months, and some patients after positive surgery, or chemotherapy, immunotherapy, and other treatments of advanced pancreatic cancer survival, are still not optimistic.

Although breakthroughs in genetic characterization have increased our knowledge of disease features and heterogeneity, the lack of real progress in PC during the last few decades has caused substantial concern (5). Given the constraints of PC therapy, novel therapeutic targets are necessary to enhance PC clinical outcomes. There are many related studies on pancreatic cancer worldwide, such as the recently identified super-enhancers such as JQ1, IBET, and SLC1A5 which can promote the EMT progression in pancreatic cancer (6–8). Current first-line therapies for advanced pancreatic cancer including gemcitabine combined with albumin-binding paclitaxel, FOLFIRINOX (fluorouracil, leucovorin, irinotecan and oxaliplatin), and modified FOLFIRINOX (9), or PARP inhibitors, such as olaparib, have been approved for germline patients with germline BRCA1 or BRCA2 mutations (10) but have not significantly improved survival. In the surgical specimens of pancreatic cancer, the pancreatic cancer tumor can be found as a solid tumor and containing a large amount of extracellular matrix in the tumor tissue, which significantly reduces the drug penetration into the tumor. It has been shown that hyperbaric oxygen significantly consumes the main components of the extracellular matrix, thus promoting the infiltration of cytotoxic T cells into the tumor parenchyma to facilitate drug penetration (11). At the same time, a prospective study mentioned that preoperative HBO pretreatment of pancreatic surgery can effectively reduce postoperative complications in patients (12). Therefore, HBO and gemcitabine may improve drug resistance and open a new chapter in the treatment of pancreatic cancer, a fatal malignant tumor, which still needs to be further explored. However, very few people can be effectively used in clinical practice. As a result, improved prognostic approaches that are both trustworthy and accurate are urgently needed to make targeted therapy more practical. The pyroptosis-related genes we studied may also provide new targets for the treatment of pancreatic cancer (13).

Pyroptosis, also known as cellular inflammatory necrosis, is a new kind of programmed cell death (14, 15). Pyroptotic cells have cellular swelling as well as many bubble-like protrusions. Under an electron microscope, pyroptotic cells may be seen forming a large number of vesicles. Following the development of these vesicles, openings form on the cell membrane, which ruptures and allows the contents to escape (16). The gasdermin family, which comprises gasdermin-A through to gasdermin-E and pejvakin, is the major executioner of pyroptosis (17). Shearing and multimerization of the gasdermin family proteins result in the cleavage of the N-terminal along with the C-terminal junctional structural domains as well as the release of activated N-terminal domains that dock to membrane lipids, cardiolipin, and phosphatidylinositol and localize into cell membrane pores (18). Cellular gasdermin family proteins have pores in the cell membrane that range from 10 to 20 nm in size, and cell contents are slowly discharged *via* membrane pores, generating increased inflammatory reactions (19, 20). Cells flatten progressively, forming 1–5-m apoptotic vesicle-like protrusions (scorched vesicles), and cells inflate gradually until the plasma membrane ruptures, exhibiting characteristics such as nuclear condensation and chromatin DNA disintegration (21, 22). Pyroptosis was first recognized as a remarkable process for fighting infection, and multiple investigations show that it is also important in tumorigenesis (23). Inflammatory vesicles, gasdermin proteins, and proinflammatory cytokines are linked with carcinogenesis, infiltration, and metastasis as pivotal elements of pyroptosis (24).

Until now, there have been many studies on prognostic models, such as recent ones identifying novel signatures for the prognosis of multiple cancers (19, 25–28). GSDMD and GSDME play significant roles in defense against intracellular pathogens and tumors. A recent study showed that GSDMD-mediated pyroptosis is closely associated with the prognosis of hepatocellular carcinoma, rectal adenocarcinoma, and cutaneous melanoma. The authors of this study highlighted the role in the antitumor immunity of GSDMD and argued for developing future drugs for activating GSDMD (29). L61H10, a thiopyran derivative of curcumin, can induce pyroptosis through the caspase-3/GSDME pathway. It has a good antitumor activity against lung cancer (30). L61H10 can promote the expression of antiapoptotic genes by regulating NF-κB in inducing the transition from apoptosis to pyroptosis. However, curcumin bioavailability *in vivo* is low, and its use for lung cancer treatment needs further research (31). Increasing the induction of tumor-related pyroptosis can reduce the tumor volume. If this move can be further studied and applied to clinical practice, it may be a crucial step in the process of human play against tumors. According to Wang et al., the bio-orthogonal system that is based on Phe-BF3 desilylation is a potent tool for chemical biology, implying that pyroptosis-triggered inflammation generates robust antitumor immunity and may synergize with checkpoint blockade (32). Therefore, the study of pyroptosis-related genes in pancreatic cancer is important for the treatment of pancreatic cancer.

It was found that application of this bio-orthogonal system to gas proteins revealed that less than 15% of tumor cells with pyroptosis were capable of clearing the entire 4T1 breast tumor graft (33). Various danger-linked signaling molecules along with cytokines are stimulated and produced when pyroptosis occurs, coupled with a severe inflammatory response as well as immune system activation (34). Some investigations have documented that pyroptosis’ powerful proinflammatory activity may influence tumor immune microenvironment modulation (35).

A considerable reduction in the number as well as activity of CD8+ T cells was linked with aberrant GSDMD expression (36). Additionally, pyroptosis plays an indispensable role in NK cell antitumor activity (37). We confirm that pyroptosis is important in tumorigenesis and antitumor processes based on our present results; nevertheless, its particular activities in PC have received less attention. As a result, we conducted a comprehensive investigation to compare the expression levels of PRGs in healthy and pancreatic adenocarcinoma tissues, investigate their prognostic worthiness, and assess the relationships of pyroptosis with the tumor immune microenvironment. We established that pyroptosis is strongly linked to tumorigenesis and that pyroptosis may cross talk with metabolites in the TME as well as particular targets on the cell membrane to execute an antitumor impact (38).

## Materials and Methods

### Dataset

The Cancer Genome Atlas (TCGA) data resource was adopted to abstract PC-clinical information, PC-RNA sequencing patterns (n = 178), and non-malignant pancreas epithelium RNA sequencing patterns (n = 4) (39). We excluded PC subjects who had no survival time, retaining 177 subjects for further research. Besides, as an external verification data set, we abstracted GSE62452 along with the GSE71729 data sets from the GEO data resource, which included 69 subjects (four cases were excluded) and 125 subjects with primary PC, respectively. Meanwhile, genes were uncovered using the GENCODE data resource (40), GPL6244, and GPL20769 annotation documents. Additionally, 33 PRGs were abstracted on the basis of previous research (41). TCGA data resource was utilized to generate mutation along with CNV data. Furthermore, the maftools program was adopted to generate the mutation frequencies of 33 PRGs in PC subjects (42).

### Construction of the PPI Network

Thirty-three PRGs were loaded into a STRING data resource (confidence = 0.900) to generate PPI networks in order to identify cross-talking genes.

### Sample Classification for Aging Patterns

We adopted the “ConsensusClusterPlus” R tool to conduct two classifications on the basis of the 33 PRGs. The optimal k value was established across all PC subjects by estimating the inflection point of the sum of squared error (SSE) according to the prognostic ARG expression. The decline rate reduced after k = i; thus, k = i was selected. Moreover, in pyroptosis profiles, we executed Kaplan–Meier survival assessment on cluster-1 and -2 groups.

### Construction of Risk Signature and Nomogram

Differential PRGs in malignant and non-malignant tissues were assessed *via* the “limma” package (|logFC| >2 along with P < 0.05). Univariate Cox regression was then adopted to filter prognostic PRGs (P < 0.05). These prognostic genes were then utilized to determine genes involved in signature creation *via* multivariate Cox coupled with LASSO regression models. To generate the model and control the complexity of LASSO regression, we employed the suitable λ. The following formula was used to determine the risk score: OS score of $\text{OS score of Risk = }\sum_{\text{i}=1}^{\text{n}}{\text{Coef}}_{\text{i}}*{\text{x}}_{\text{i}}$ (43). Besides, for verification, we implemented Kaplan–Meier survival assessment, ROC analysis, and a calibration curve. For clinical utility, the comparative value (2^-Δct^) was calculated from qRT-PCR results and used for score calculation, and the score was further standardized and simplified to generate a riskscore (44). The riskscore was calculated as follows: Riskscore (qRT-PCR) =(score-min)/max.

### Functional Enrichment Analysis

The “ggplot2” and “clusterProfiler” R tools were adopted to execute enrichment analysis in 33 PRGs. The data of the “clusterProfiler” tool were utilized to conduct gene ontology (GO) assessment along with the Kyoto Encyclopedia of Genes and Genomes (KEGG) assessment. Moreover, GSEA enrichment analysis was done in numerous risk groups differentiated by risk signature (42).

### Comprehensive Immune, TMB, and MSI Analyses

We adopted the ssGSEA approach to quantify the abundance of immune cells in distinct risk groups separated by risk signature while investigating disparities in immune cell invasion. We also adopted the Timer algorithm to assess the immune cell correlation. More critically, we assessed the levels of TMB along with MSI expressions in each risk signature gene.

### Drug Sensitivity Analysis

Chemotherapeutic drugs were abstracted from the Genomics of Drug Sensitivity in Cancer (GDSC) data resource, and IC50 was computed *via* the “pRRophetic” R tool (42, 43, 45).

### Cell Culture and Real-Time PCR

A total of 14 tumor tissue samples and 10 normal tissue samples were obtained from PC patients who underwent tumor resection. All tissue samples were collected from the Affiliated Hospital of Nantong University with the approval by the Medical Ethics Committee. The National Infrastructure of Cell Line Resource provided a human normal pancreatic duct epithelial cell line (HPDE6-C7) and human pancreatic cancer cells (PANC-1, BXPC-3, CFPAC-1). They were grown in DMEM or RPMI-1640 enriched with 10% FBS, along with 1% penicillin/streptomycin, and incubated at 37°C in a humidified incubator containing 5% CO_2_. Total RNA was extracted from Hp, PANC, BX, and CF cells with TRIzol (Invitrogen). The complement DNA (cDNA) was prepared with a RevertAid First Strand cDNA Synthesis Kit (TaKaRa Bio, Kusatsu, Japan). All quantitative real-time PCRs were carried out in triplicate with SYBR Master Mix (TaKaRa Bio) on a LightCycler 480 II instrument (Roche, Basel, Switzerland). The primers were used as follows:

RT-IL-18-F:TCTTCATTGACCAAGGAAATCGG,
RT-IL-18-R:TCCGGGGTGCATTATCTCTAC;
RT-TNF-F:ACCCTCACACTCACAAACCA,
RT-TNF-R:ATAGCAAATCGGCTGACGGT;
RT-NLRP1-F:CCCCATCCCTCTGAGCTAC,
RT-NLRP1-R:ACTTAACAGGCCCAATAGGAA;
RT-CASP4-F:TGGCAGAAGGCAACCACAGAA,
RT-CASP4-R:TTTGTTCCACCAAGTTATCC.

## Results

### Differential Expression and Mutation Landscape of PRGs in PC Patients

**Figure 1:**
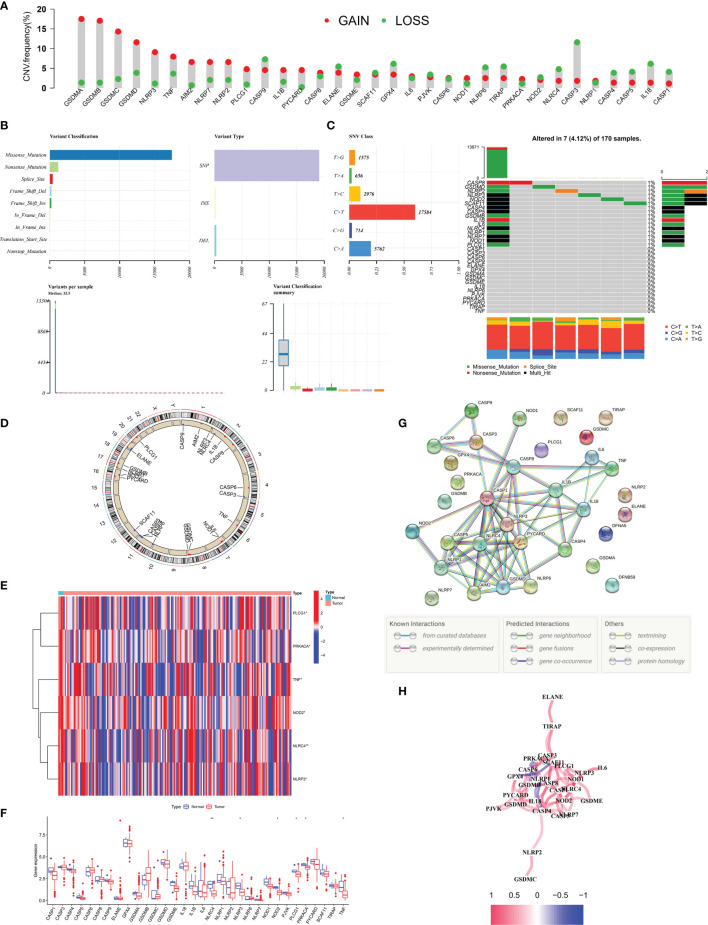
Landscape of 33 PRGs in the PC cohort. **(A)** The CNV alteration of 33 PRGs in the PC cohort. The height of the column represents the alteration frequency. **(B, C)** The mutation frequency along with the classification of 33 PRGs in PC. PRG: pyroptosis-related gene, PC: pancreatic cancer, SNP: single-nucleotide polymorphism, INS: insertion, and DEL: deletion. **(D)** The position of 33 PRGs on the chromosome. **(E)** Heatmap (blue: low expression level; red: high expression level) of PRGs between normal (N, brilliant blue) and tumor tissues (T, red). **(F)** Comparison of the gene expression of 33 types of PRGs between normal and tumor tissues in TCGA cohort. **(G)** PPI network illustrating the cross talks of PRGS (cross talk score = 0.9). **(H)** The association network of PRGs (red line: positive association; blue line: negative relationship). The depth of colors reflects the strength of relevance). *P < 0.05; **P < 0.01.

We explored the mutation landscape of 33 PRGs that could be annotated in TCGA-PAAD cohort. First, we revealed the copy number variation (CNV) of 33 PRGs in PC patients (**Figure 1A**). All PRGs had copy number amplification or deletion, among which GSDMA showed the highest amplification frequency and CASP3 revealed the highest loss frequency. Mutations were found in 7 (4.12%) of 170 PC samples, with most genes mutating at about 1% (**Figure 1B**). In the assessment of the incidence of somatic mutations in 33 PRGs, missense mutation was the most common variation classification. Single-nucleotide polymorphism (SNP) was the most common variation type, and C > T was dominant in the single-nucleotide variant (SNV) (**Figure 1C**). In addition, we identified the corresponding position of 33 PRGs on the chromosomes (**Figure 1D**). Meanwhile, we compared the expression levels of PRGs in TCGA-PAAD cohort, and the heatmap revealed six differentially expressed PRGs, including PLCG1, PRKACA, TNF, NOD2, NLRC4, and NLRP3 (**Figure 1E**). Interestingly, the boxplot demonstrated that the above six genes were downregulated in tumor tissue (**Figure 1F**). To further explore the cross talk between PRGs, we conducted PPI analysis, and the minimum confidence required for analysis was set at 0.9 (**Figure 1G**). Finally, we showed a correlation network of 33 PRGs (red: positive correlation; blue: negative correlation), and the results revealed more red line segments than blue line segments (**Figure 1H**).

### Pyroptosis Patterns in PC Patients

**Figure 2:**
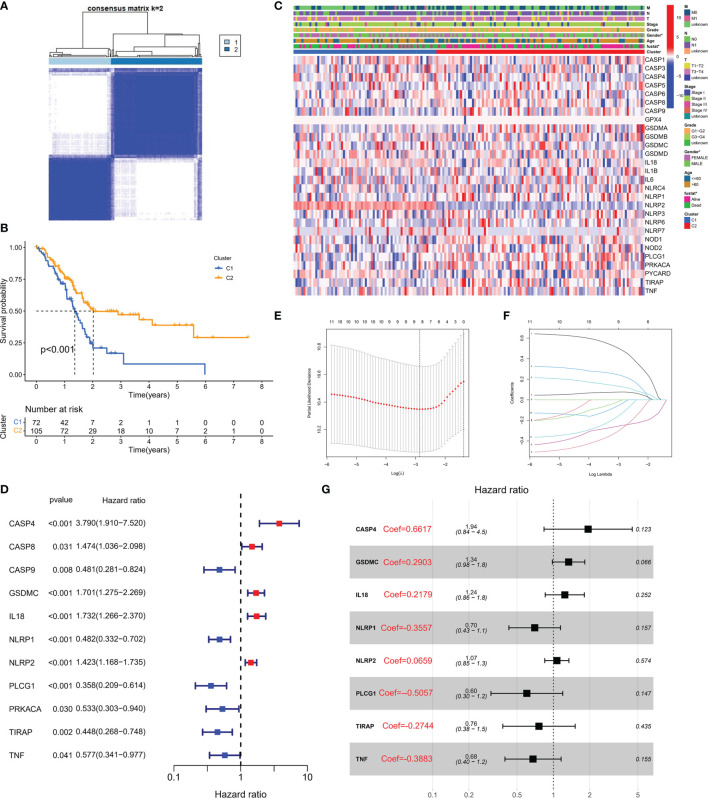
Molecular subtypes and risk status based on PRG expression. **(A)** According to the consensus clustering matrix (k = 2), 190 PC subjects were stratified into two groups. **(B)** Kaplan–Meier curves for the two clusters. **(C)** The heatmap along with the clinicopathologic characteristics of the two clusters established using these DEGs and clinical variables. (T refers to tumor, N refers to lymph node, and M refers to distant metastasis). T1 means that the tumor is less than 2 cm, T2 is the tumor in 2–4 cm, T3 is the tumor larger than 4 cm, and T4 is the tumor regardless of size but invades the celiac artery, superior mesenteric artery, or common hepatic artery. N refers to lymph nodes, N1 refers to regional metastases in one to three lymph nodes, N2 refers to regional metastases in four or more lymph nodes, and M1 refers to distant metastasis of the tumor. On this basis, the combination of the three TNM indicators was used to draw a specific stage (stage). G1, G2, and G3 are the degree of tumor differentiation (G1: highly differentiated; G2: moderately differentiated; G3: poorly differentiated). **(D)** Univariate Cox regression analysis of PRGs in TCGA cohort. **(E)** Cross-verification was adopted to fine-tune the selection of parameters in LASSO regression. **(F)** LASSO regression of 11 genes linked to PC. **(G)** Multivariate Cox regression analysis of PRGs in TCGA cohort.

To explore the association between PRG expression and PC occurrence, we performed consensus clustering in PC patients and found that when k = 2, the intra-group correlation was the highest, while the inter-group correlation was low (**Figure 2A**). It indicated that PC patients could be well divided into two groups according to 33 PRGs. According to the above algorithm, all patients were classified into subgroups C1 and C2. In addition, the overall survival (OS) between the two subgroups was also compared; excitingly, the survival time of the C2 subgroup was remarkably shorter than that of the C1 subgroup (P< 0.001), as displayed in **Figure 2B**. Heatmap results demonstrated remarkable differences in age and survival status between the subtypes (P< 0.05), as depicted in **Figure 2C**. To clarify other biological functions of PRGs, except for regulating pyroptosis, we conducted GO and KEGG enrichment analyses. GO enrichment analysis revealed that PRGs were mainly involved in regulating cytokine production, interleukin-1 function, and other biological processes (**Table S1**). In addition, KEGG analysis demonstrated that PRGs were mainly involved in the NOD-like receptor signaling pathway, *Salmonella* infection, and other cascades (**Table S2**).

### A PRG-Based Risk Stratification System Was Developed in PC Patients

**Figure 3:**
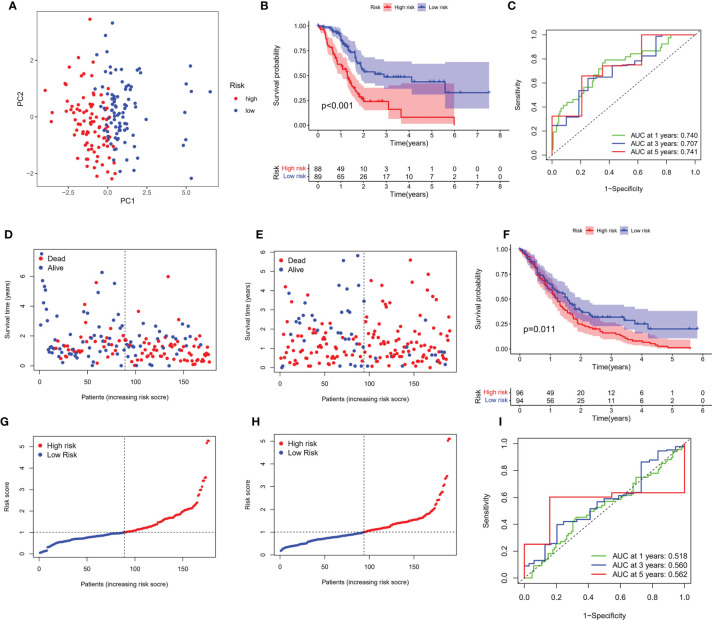
Verification of risk score. **(A)** PCA map for PC in TCGA cohort. **(B)** Kaplan–Meier curves for comparison of PC risks between low-PC and high-PC risk groups. **(C)** ROC curves illustrating the risk score prediction efficiency. **(D)** Each patient’s survival rate is shown in TCGA cohort (low-PC risk cluster: on the left side of the dotted line; high-PC risk class: on the right side of the dotted line). **(E)** The survival rate for each patient in TCGA cohort (low-PC risk group: on the left side of the dotted line; high-PC risk class: on the right side of the dotted line). Patient distribution on the basis of the risk score. **(F)** Each patient’s survival rate is shown in GEO cohort. **(G)** The survival rate for each patient in TCGA cohort. **(H)** Kaplan–Meier curves for comparison of PC risks between low-PC and high-PC risk groups. **(I)** ROC curves illustrating the risk score prediction efficiency.

Transcriptome data and survival information were matched, and TCGA-PAAD cohort eventually included 177 patients for follow-up analysis. Univariate Cox regression assessment was adopted for preliminary screening of prognostic genes, and 11 genes were identified (**Figure 2D**). Risk stratification systems containing 8-PRGs were constructed based on the optimal λ values by LASSO (**Figures 2E, F**) and multivariate Cox regression assessment (**Figure 2G**). The risk score was computed as follows: risk score = (0.6617*CASP4-exp.) + (0.2903*GSDMC-exp.) + (0.2719*IL18-exp.) + (0.3557*NLRP1-exp.) + (0.0659*NLRP2-exp.) + (-0.5057*PLCG1 exp.) + (-0.2744*TIRAP-exp.) + (-0.3883*TNF exp.). According to the median score computed by the formula, 177 PC patients were divided into low- and high-risk subgroups. PCA revealed that different risk patients were well divided into two groups (**Figure 3A**). In contrast with the low-PC risk group, subjects in the high-PC risk group had shorter survival time and remarkable differences in OS time (**Figure 3B**). Time-dependent receiver operating characteristic (ROC) assessment was adopted to assess the sensitivity along with the specificity of the risk stratification system. We found that the areas under the ROC curve (AUC) were 0.740, 0.707, and 0.741 at 1, 3, and 5 years, respectively (**Figure 3C**). In addition, the risk profile also showed the same results (**Figures 3D, E**). Kaplan–Meier analysis was performed on GEO and TCGA cohorts of each PRG participating in the risk stratification system (**Figure S1**).

### External Validation

An external validation cohort of 190 PC patients was abstracted from the GEO database (GSE62452 and GSE71729). The data of the two datasets were standardized by the “sva” package before further analysis. On the basis of the median risk score in TCGA cohort, 190 patients in the GEO cohort were stratified into the low-risk group (94 patients) and high-risk group (96 patients), as presented in **Figures 3F, G**. Kaplan–Meier analysis demonstrated that the survival difference between the two subgroups was statistically significant (P = 0.011, **Figure 3H**). ROC curve analysis of the GEO cohort indicated that our risk stratification system had a good prediction effect (AUC values of 1, 3, and 5 years were 0.518, 0.560, and 0.562, respectively), as depicted in **Figure 3I**.

### Construction Nomogram Based on Independent Prognostic Factors

**Figure 4:**
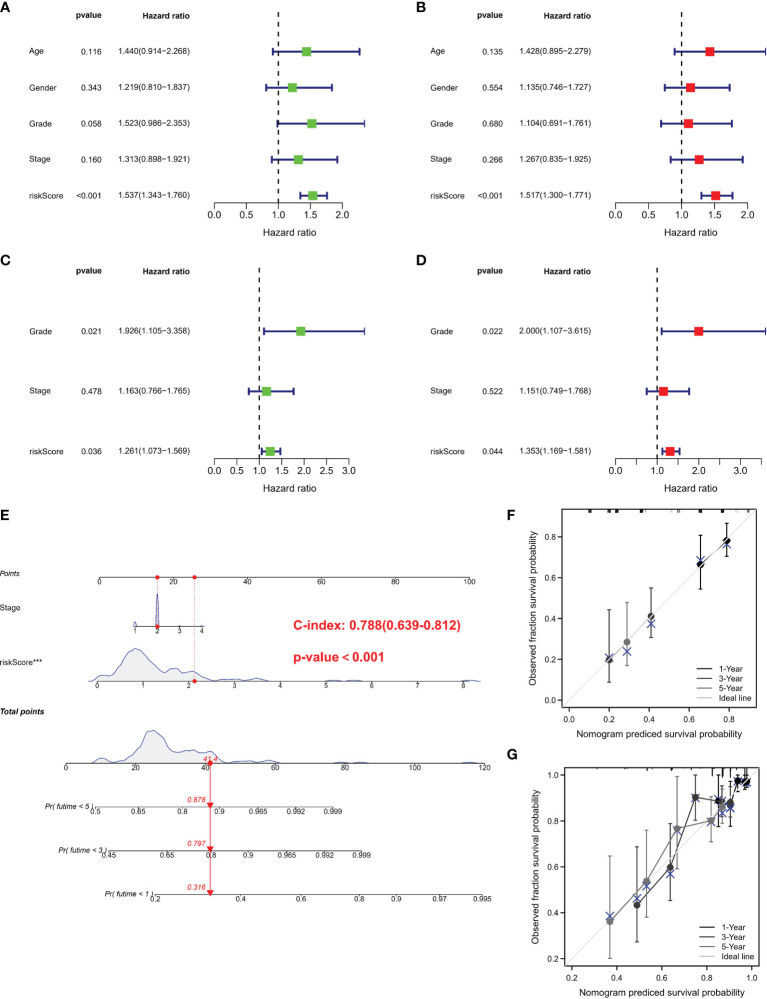
Independent prognostic validation and construction of nomogram. **(A)** Univariate assessment for TCGA cohort (grade: degree of tumor differentiation, G1 to G3; stage: the size of the primary tumor and the degree to which the cancer has spread in the patient’s body, I to IV). **(B)** Multivariate analysis for TCGA cohort. **(C)** Univariate analysis for the GEO cohort. **(D)** Multivariate analysis for the GEO cohort. **(C, F)** Heatmap (green: low expression; red: high expression) for the connections between clinicopathologic features and risk groups. **(E)** Nomogram. **(F, G)** Nomogram to predict 1-, 3-, and 5-year overall survival rates of PC patients. Calibration curve for the overall survival nomogram model in the discovery group. A dashed diagonal line represents the ideal nomogram. PRG, pyroptosis-related gene; PC, pancreatic cancer.

Univariate and multivariate Cox regressions were adopted to assess whether the risk score was an independent prognostic factor in PC subjects. Univariate Cox regression data revealed that risk score was a risk factor in predicting poor survival in TCGA (**Figure 4A**) and GEO cohorts (**Figure 4C**) (HR =1.537, HR: 1.261). Multivariate analysis also illustrated that risk score was an independent prognostic factor (HR = 1.517, HR: 1.353) (**Figures 4B, D**). We combined the clinical characteristics commonly employed in clinical work to establish a nomogram to estimate survival probability (**Figure 4E**). Calibration curves illustrated that the prediction of overall survival was relatively linked to the standard curve in the two cohorts (**Figures 4F, G**).

### Comparison of Immunity Between Subgroups

**Figure 5:**
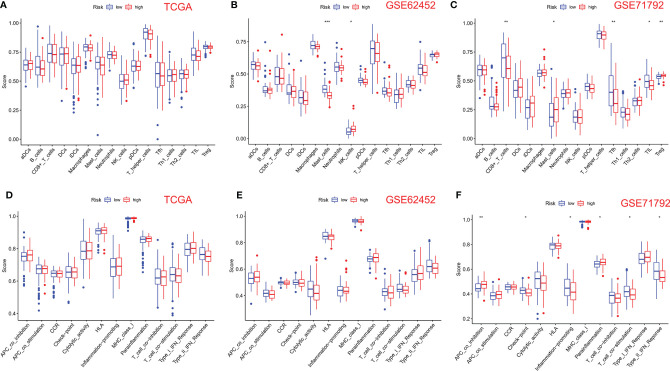
Comparison of ssGSEA scores for immune cells and cascades. **(A, D)** Comparison of enrichment scores of 16 types of immune cells and 13 immune-related cascades between low- (blue box) and high-risk (red box) groups in TCGA cohort. **(B, E)** Comparison of tumor immunity between low- (blue box) and high-risk (red box) groups in the GSE62452 cohort. **(C, F)** Comparison of tumor immunity between low- (blue box) and high-risk (red box) groups in the GSE71792 cohort. P-values were shown as follows: ns, not significant; *P < 0.05; **P < 0.01; ***P < 0.001.

We use the ssGSEA algorithm to further compare the enrichment score of 16 immune cells and 13 immune-linked cascades in low- and high-risk subgroups. Interestingly, there was no correlation about immune cells and immune-related cascades in each group in TCGA cohort (**Figures 5A, D**). However, mast and NK cells were lower in high-risk patients than in low-risk patients in the GSE62452 cohort (**Figure 5B**). TCGA and GSE62452 cohorts also showed no statistical differences in immune function (**Figure 5E**). In the GSE71792 cohort, there were five types of immune cells and six types of immune function that differed remarkably between groups (**Figures 5C, F**). In addition, we elucidated the immune infiltration of prognostic PRGs in the TIMER database (**Figure S2**).

### TMB, MSI, and Drug Sensitivity Analyses

TMB can be used as a biomarker to predict the efficacy of immunotherapy for PC, and MSI is also considered a marker for cancer immunotherapy. To determine whether 8-PRGs involved in the risk signature can also be used as biomarkers for drug selection, we analyzed the correlation between 8-PRGs and drug, MSI, and TMB. The results indicated that MSI was negatively linked with IL18 and TNF (**Figure S3**). TMB was negatively linked with NLRP1 and positively with IL18 (**Figure S4**). Herein, drug sensitivity assessment demonstrated that IL18 expression was negatively linked to some or most drugs in the tumor therapeutic response Portal database (**Table S3**).

### Validation of PRGs in Clinical Samples and Cell Lines

**Figure 6:**
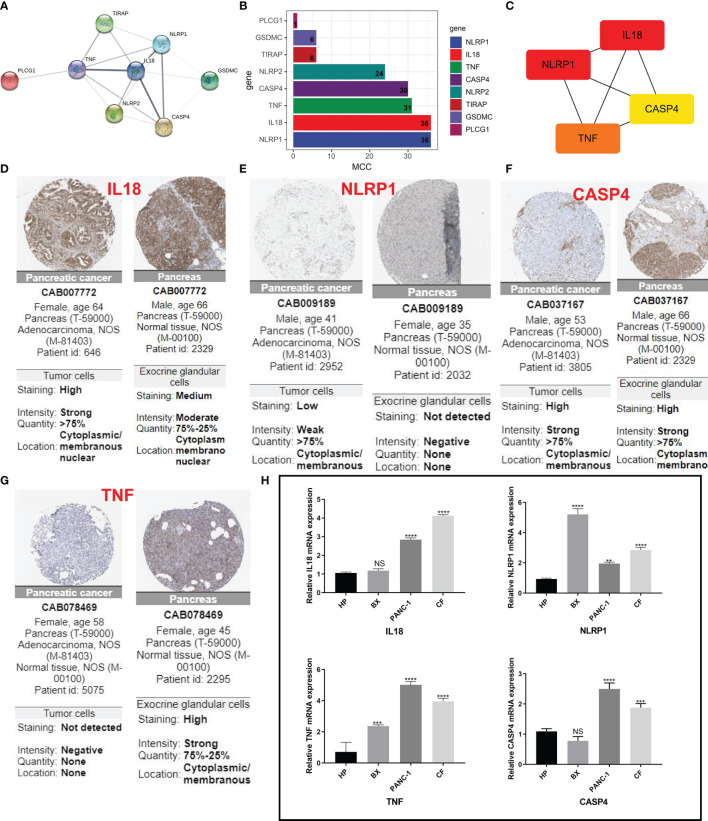
IHC and immunohistochemical verification. **(A)** PPI network showing the cross talks of eight pyroptosis-related genes. **(B, C)** The MCC algorithm is used to calculate the expression score of these eight genes in pancreatic cancer and links between the four genes with higher expression scores. **(D–G)** HPA data resource was also used to explore the expression of these four markers in pancreatic cancer tissue samples and normal tissues. **(H)** The mRNA levels from different cells as determined by real-time PCR analysis. **P < 0.01; ***P < 0.001; **** < 0.0001. NS means not significant.

**Figure 7:**
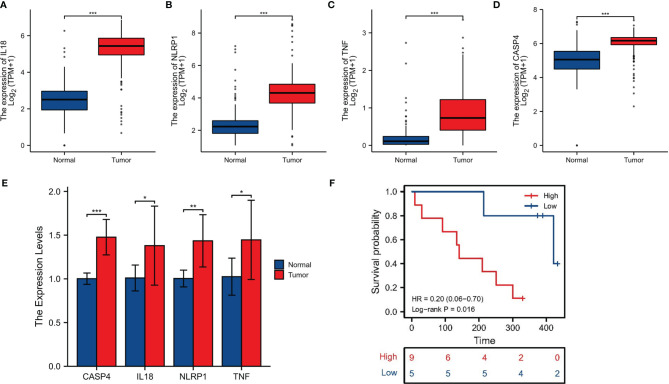
Clinical sample validation. **(A–D)** The expression of four signature-associated genes between tumor tissues and normal tissues on basis of the GTEx database and TCGA database. **(E)** qRT-PCR of the expression of four signature-associated genes in clinical samples. **(F)** Survival analysis. *p < 0.05; **p < 0.01; ***P < 0.001.

For further screening, we calculated the MCC score in the PPI network and identified NLRP1, IL18, TNF, and CASP4 as the hub genes in the protein level (**Figures 6A–C**). Meanwhile, the IHC imaging from the HPA database further confirmed the expression in different tissues (**Figures 6D–G**). Similar to the results in **Figure 1F**, the protein expression in different tissues was also significantly different. RT-qPCR assessment was performed in four cell lines, comprising three tumor cell lines along with a human immortalized normal ductal epithelial cell line (**Figure 6H**). Compared with the human immortalized normal ductal epithelial cell line, the expression of NLRP1 and TNF in tumor cell lines was remarkably higher, the expression of NLRP1 was the highest in BX cell lines, and the expression of TNF was the highest in PANC-1 cell lines. IL18 and CASP4 expressions were not observed in the Bx cell lines. In CF cell lines, the expression of these four genes was remarkably higher. For clinical samples, we used the GTEx database, TCGA database, and qRT-PCR assay to analyze the expression levels of four PRGs. As shown in **Figures 7A–E**, tumor tissues showed obviously higher expression levels of NLRP1, IL18, TNF, and CASP4 than did the normal tissues. Importantly, survival analysis also showed that the high-risk group had a shorter survival time in our hospital cohort (P < 0.05) (**Figure 7F**). The experimental findings presented above were congruent with the predictions made by bioinformatics approaches.

## Discussion

Pyroptosis is a recently identified kind of programmed cell death that has a dual role in cancer progress and therapeutic methods. Pyroptosis is known to be associated with tumor suppression. Activation of the pyroptosis pathway can effectively inhibit tumor progression, and some cancer cells escape tumor suppression also by escaping the pyroptosis pathway (46). Nevertheless, the function of PRG in PC remains unclear, and our work was designed to elucidate its role. We focused on defining the expression and prognostic worthiness of PRGs in PC. When comparing PC to normal tissues, we reported an improvement in IL18 expression and a reduction in TNF expression. A prognosis study found a low survival rate in PC patients with elevated NLRP1, NLRP2, IL18, and CASP4 expressions. These findings were consistent with previous reports.

As an inflammasome of NLRP3, IL-18 can accelerate atherosclerosis in mice (47). TNF is one of potential inducers of necroptosis in PDA (48). NLRP1 has been shown to predispose individuals to multiple self-healing palmoplantar carcinoma (MSPC) and familial keratosis lichenoides chronica (FKLC) (49). CASP4 and IL-18 are both involved in the pathogenesis of alcoholic hepatitis (AH) but has opposite effects in the pathogenesis. A cross-analysis has identified CASP4 as a commonly up-regulated gene known to be involved in the non-canonical inflammasome pathway. CASP4 deficiency reduces the severity of AH. Conversely, the deficiency of interleukin-18, the key antimicrobial cytokine, aggravates hepatic bacterial load, GSDMD activation, and AH (50). Additionally, we performed a functional enrichment analysis of PRGs and revealed that these 33 PRGs were mainly involved in the NOD-like receptor signaling pathway, TNF signaling pathway, regulating inflammatory response, pyroptosis, apoptosis, and Toll-like receptor signaling pathway. These findings suggested that these 33 PRGs may also be critical in PC oncogenesis and progression. Nowadays, there have also been many studies on the treatment of pancreatic cancer. A study of nutrient innervation in pancreatic cancer found that TRK-NGF inhibitors could interfere with the axon–nerve axis and reduce the nutrient supply of PDAC to reduce tumor recurrence (38, 51). The macrophage phenotypic switch-related signature could predict metastasis and survival in pancreatic cancer patients (52). In addition, CXC chemokine expression and their biological functions in pancreatic cancer may demonstrate good performance in PC patient prognosis and immunotherapeutic target therapy prediction (53). A prognostic gene model based on eight prognostic PRGs (CASP4, GSDMC, IL-18, NLRP1, NLRP2, PLCG1, TIRAP, and TNF) was constructed using LASSO Cox regression analysis and could predict the overall survival of PC patients with medium-to-high accuracy. In comparison to an ideal model, a predictive nomogram revealed that 3- and 5-year overall survival rates in the entire cohort could be predicted relatively well. Our research was the first to identify a pyroptosis-related prognostic gene signature for PC, expanding the number of options for prognostic prediction in the disease.

Our study has some limitations. Due to the extremely low survival rate for pancreatic cancer, our risk stratification system is not predictive in external experiments. Other limitations include the relatively small number of normal samples from TCGA database, and the hypothesis did not use animal models to validate. To be precise, these factors must be verified in further investigations. Therefore, in the future, we will create animal models to test these hypotheses further.

## Data Availability Statement

The datasets presented in this study can be found in online repositories. The names of the repository/repositories and accession number(s) can be found in the article/**Supplementary Material**.

## Ethics Statement

The study was authorized by the Ethical Committee of Affiliated Hospital of Nantong University (2022-L096).

## Author Contributions

JZ and XY conceived of and designed the study. DK were responsible for the materials and supplement experiments. JZ drafted the manuscript. GZ critically revised the manuscript. All authors contributed to the article and approved the submitted version.

## Funding

This study was funded by the National Natural Science Foundation of China (81572397).

## Conflict of Interest

The authors declare that the research was conducted in the absence of any commercial or financial relationships that could be construed as a potential conflict of interest.

## Publisher’s Note

All claims expressed in this article are solely those of the authors and do not necessarily represent those of their affiliated organizations, or those of the publisher, the editors and the reviewers. Any product that may be evaluated in this article, or claim that may be made by its manufacturer, is not guaranteed or endorsed by the publisher.

## Supplementary Material

Supplementary Table 1 Result of GO analysis.

Supplementary Table 2 Result of KEGG analysis.

Supplementary Table 3 Results of drug correlation analysis.

Supplementary Figure 1 Survival analysis of signature-associated genes in different data sets.

Supplementary Figure 2 The relationship of eight prognostic PRGs with immune infiltration.

Supplementary Figure 3 Correlation analysis between MSI and PRG in PC.

Supplementary Figure 4 Correlation analysis between TMB and PRG in PC.
