# Supplementary material for: Exploring the Potential of Pyroptosis-Related Genes in Predicting Prognosis and Immunological Characteristics of Pancreatic Cancer From the Perspective of Genome and Transcriptome
Source: Front Oncol. 2022 Jun 16;12:932786. doi: 10.3389/fonc.2022.932786 (PMC9243448; doi:10.3389/fonc.2022.932786)
Supplement: Supplementary Table 3 — Results of drug correlation analysis. [file Table_3.docx]

| Gene | Drug | cor | pvalue |
| --- | --- | --- | --- |
| TNF | Nelarabine | 0.7460 | 0.0000 |
| GSDMC | Ixazomib citrate | -0.5760 | 0.0000 |
| IL18 | Pipamperone | -0.5090 | 0.0000 |
| IL18 | Bortezomib | -0.5088 | 0.0000 |
| TNF | Dexamethasone Decadron | 0.5036 | 0.0000 |
| GSDMC | Midostaurin | -0.4919 | 0.0001 |
| TNF | FlupheNAzine | 0.4864 | 0.0001 |
| NLRP1 | PoNAtinib | 0.4734 | 0.0001 |
| GSDMC | Bortezomib | -0.4636 | 0.0002 |
| IL18 | Actinomycin D | -0.4490 | 0.0003 |
| IL18 | Estramustine | -0.4407 | 0.0004 |
| IL18 | Vemurafenib | -0.4394 | 0.0004 |
| GSDMC | pralatrexate | -0.4361 | 0.0005 |
| NLRP1 | Lenvatinib | 0.4272 | 0.0007 |
| IL18 | Vinblastine | -0.4024 | 0.0014 |
| IL18 | Raloxifene | -0.3963 | 0.0017 |
| IL18 | Arsenic trioxide | -0.3948 | 0.0018 |
| IL18 | Lomustine | -0.3894 | 0.0021 |
| IL18 | Carfilzomib | -0.3880 | 0.0022 |
| IL18 | Carmustine | -0.3823 | 0.0026 |
| TNF | Arsenic trioxide | 0.3810 | 0.0027 |
| IL18 | Depsipeptide | -0.3801 | 0.0027 |
| GSDMC | Vismodegib | -0.3795 | 0.0028 |
| GSDMC | Gefitinib | 0.3790 | 0.0028 |
| IL18 | Ixazomib citrate | -0.3776 | 0.0029 |
| IL18 | Sulfatinib | -0.3732 | 0.0033 |
| PLCG1 | Nelarabine | 0.3701 | 0.0036 |
| GSDMC | Vincristine | -0.3690 | 0.0037 |
| IL18 | Paclitaxel | -0.3684 | 0.0038 |
| NLRP1 | ZoledroNAte | 0.3651 | 0.0041 |
| TNF | Fludarabine | 0.3609 | 0.0046 |
| IL18 | VINORELBINE | -0.3605 | 0.0047 |
| IL18 | Mithramycin | -0.3591 | 0.0048 |
| IL18 | Dabrafenib | -0.3542 | 0.0055 |
| IL18 | Homoharringtonine | -0.3499 | 0.0061 |
| CASP4 | Fulvestrant | -0.3449 | 0.0070 |
| IL18 | Vincristine | -0.3420 | 0.0075 |
| IL18 | Vinorelbine | -0.3286 | 0.0104 |
| TNF | Cyclophosphamide | 0.3281 | 0.0105 |
| IL18 | Doxorubicin | -0.3237 | 0.0116 |
| NLRP1 | Raltitrexed | 0.3228 | 0.0119 |
| IL18 | ETHINYL ESTRADIOL | -0.3217 | 0.0122 |
| IL18 | ARSENIC TRIOXIDE | -0.3216 | 0.0122 |
| NLRP1 | Dasatinib | 0.3190 | 0.0130 |
| IL18 | Irofulven | 0.3149 | 0.0142 |
| IL18 | Epirubicin | -0.3143 | 0.0145 |
| NLRP1 | Bleomycin | 0.3135 | 0.0147 |
| CASP4 | Homoharringtonine | -0.3101 | 0.0159 |
| NLRP1 | Simvastatin | 0.3087 | 0.0164 |
| IL18 | Teniposide | -0.3084 | 0.0165 |
| NLRP1 | Axitinib | 0.3081 | 0.0166 |
| IL18 | Tamoxifen | -0.3051 | 0.0178 |
| GSDMC | Dacomitinib | 0.2977 | 0.0209 |
| IL18 | Tegafur | -0.2965 | 0.0214 |
| IL18 | Crizotinib | -0.2962 | 0.0216 |
| NLRP1 | Vinorelbine | -0.2949 | 0.0222 |
| TIRAP | Palbociclib | -0.2921 | 0.0235 |
| PLCG1 | LDK-378 | -0.2894 | 0.0249 |
| IL18 | Afatinib | 0.2887 | 0.0253 |
| NLRP1 | Tamoxifen | -0.2872 | 0.0261 |
| NLRP1 | DACARBAZINE | 0.2842 | 0.0278 |
| IL18 | Ixabepilone | -0.2827 | 0.0286 |
| IL18 | Encorafenib | -0.2827 | 0.0286 |
| NLRP1 | Pazopanib | 0.2822 | 0.0289 |
| IL18 | Dacomitinib | 0.2811 | 0.0296 |
| IL18 | Abiraterone | -0.2784 | 0.0312 |
| PLCG1 | RAPAMYCIN | 0.2767 | 0.0323 |
| IL18 | Erlotinib | 0.2731 | 0.0348 |
| IL18 | Etoposide | -0.2720 | 0.0355 |
| IL18 | Nilotinib | -0.2709 | 0.0363 |
| PLCG1 | brigatinib | -0.2708 | 0.0363 |
| TNF | Hydroxyurea | 0.2702 | 0.0368 |
| GSDMC | Idarubicin | -0.2693 | 0.0375 |
| IL18 | Eribulin mesilate | -0.2674 | 0.0388 |
| TIRAP | Midostaurin | -0.2672 | 0.0391 |
| GSDMC | Carmustine | -0.2666 | 0.0395 |
| TIRAP | DACARBAZINE | -0.2665 | 0.0396 |
| NLRP1 | Imatinib | 0.2655 | 0.0403 |
| GSDMC | DAUNORUBICIN | -0.2654 | 0.0404 |
| GSDMC | Erlotinib | 0.2636 | 0.0419 |
| PLCG1 | Fludarabine | 0.2633 | 0.0421 |
| TIRAP | Tegafur | 0.2601 | 0.0447 |
| TIRAP | ZoledroNAte | -0.2598 | 0.0450 |
| CASP4 | Doxorubicin | -0.2590 | 0.0457 |
| NLRP1 | MITOXANTRONE | 0.2590 | 0.0457 |
| GSDMC | Bisacodyl, active ingredient of Viraplex | 0.2582 | 0.0464 |
| GSDMC | Pazopanib | -0.2580 | 0.0466 |
| TNF | DACARBAZINE | 0.2568 | 0.0476 |
