# Supplementary figures and images for: Exploring the Potential of Pyroptosis-Related Genes in Predicting Prognosis and Immunological Characteristics of Pancreatic Cancer From the Perspective of Genome and Transcriptome
Source: Front Oncol. 2022 Jun 16;12:932786. doi: 10.3389/fonc.2022.932786 (PMC9243448; doi:10.3389/fonc.2022.932786)

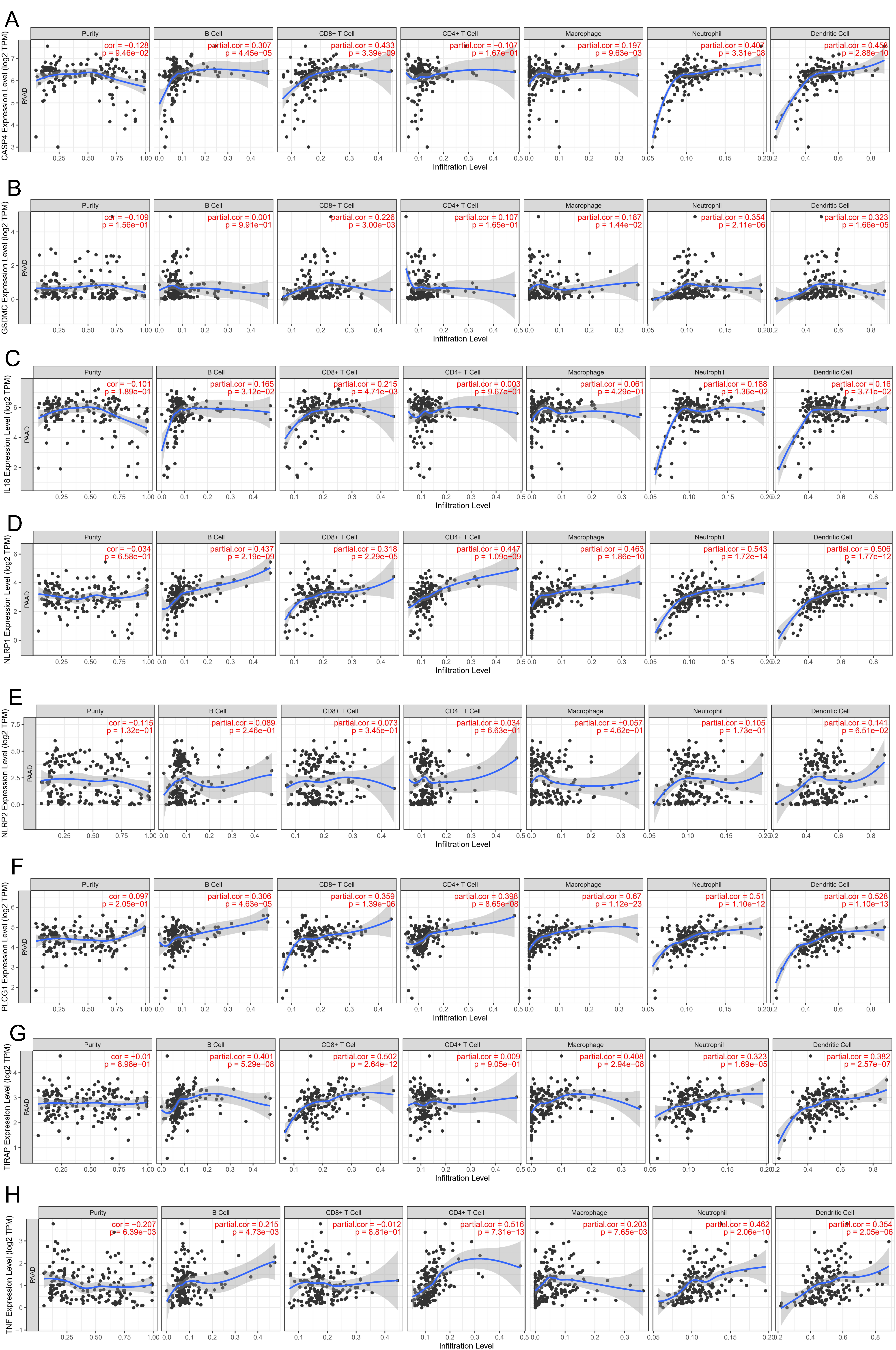

Supplement: Supplementary Figure 2 — The relationship of eight prognostic PRGs with immune infiltration. [file Image_2.tiff]

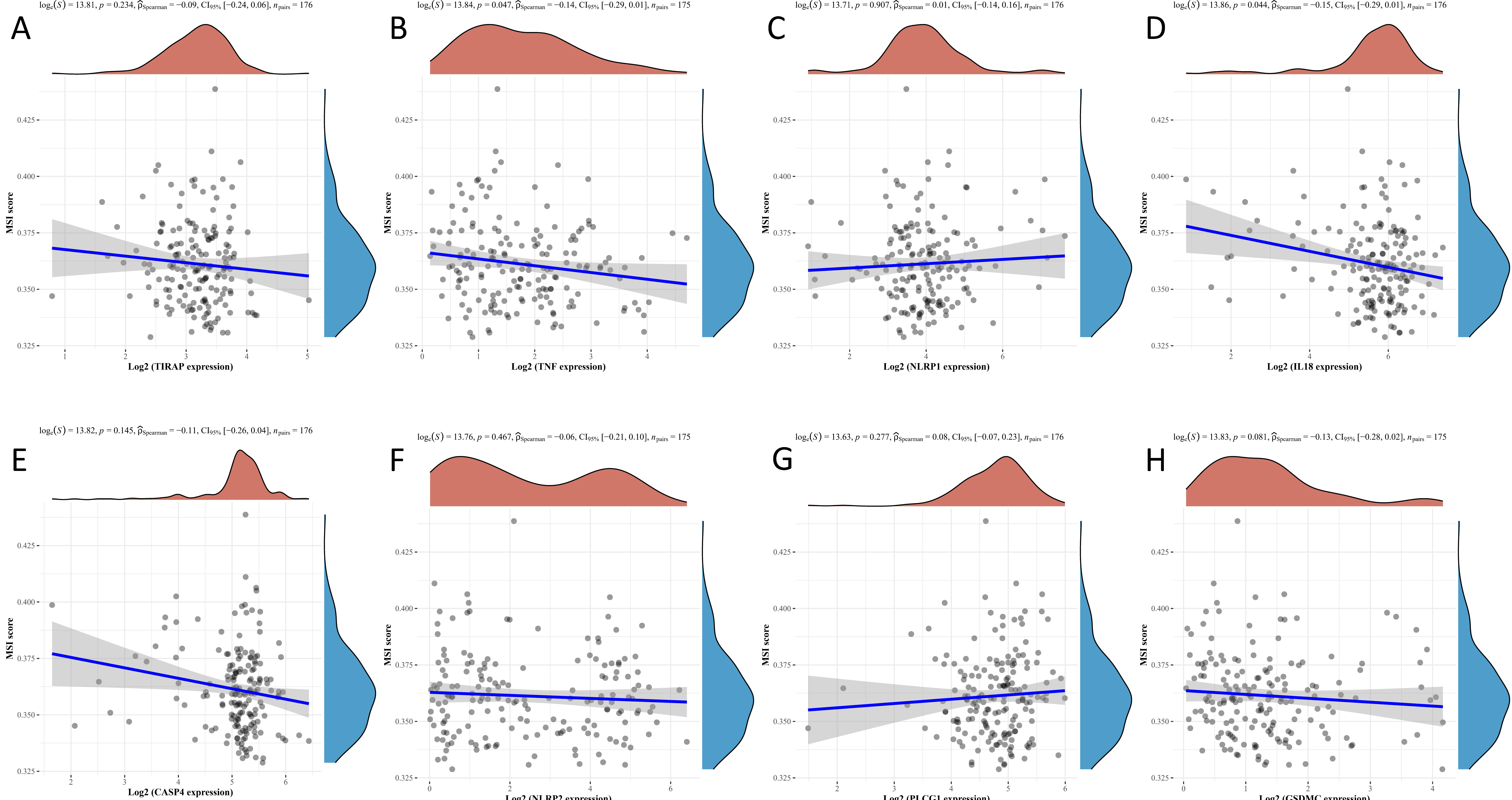

Supplement: Supplementary Figure 3 — Correlation analysis between MSI and PRG in PC. [file Image_3.tiff]

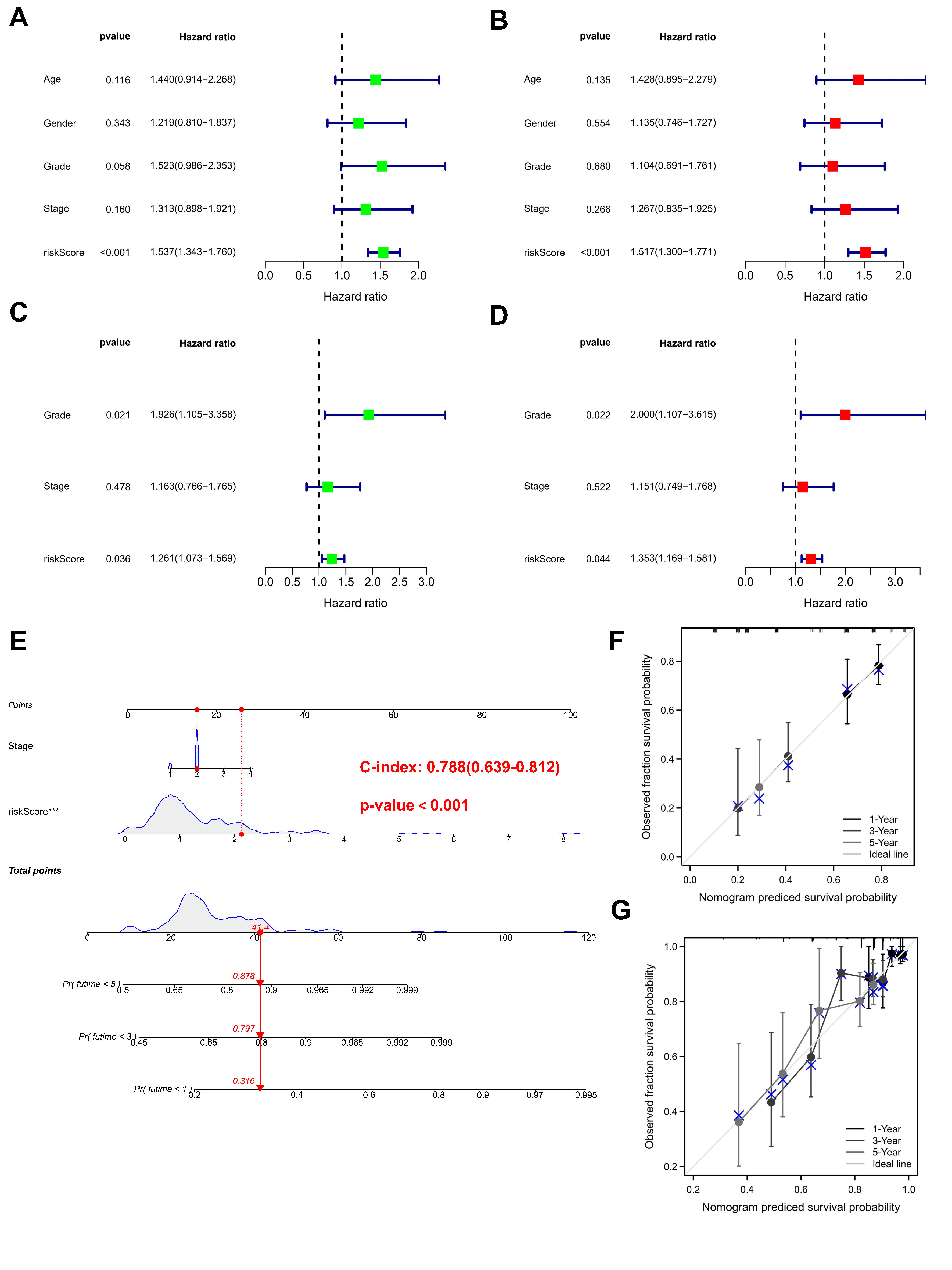

Supplement: Supplementary Figure 4 — Correlation analysis between TMB and PRG in PC. [file Image_4.tiff]
